# Exploring the Role of Brain Oscillations in Speech Perception in Noise: Intelligibility of Isochronously Retimed Speech

**DOI:** 10.3389/fnhum.2016.00430

**Published:** 2016-08-31

**Authors:** Vincent Aubanel, Chris Davis, Jeesun Kim

**Affiliations:** MARCS Institute for Brain, Behaviour and Development, Western Sydney UniversityPenrith, NSW, Australia

**Keywords:** brain oscillations, speech intelligibility, temporal modification, isochrony, syllable

## Abstract

A growing body of evidence shows that brain oscillations track speech. This mechanism is thought to maximize processing efficiency by allocating resources to important speech information, effectively parsing speech into units of appropriate granularity for further decoding. However, some aspects of this mechanism remain unclear. First, while periodicity is an intrinsic property of this physiological mechanism, speech is only quasi-periodic, so it is not clear whether periodicity would present an advantage in processing. Second, it is still a matter of debate which aspect of speech triggers or maintains cortical entrainment, from bottom-up cues such as fluctuations of the amplitude envelope of speech to higher level linguistic cues such as syntactic structure. We present data from a behavioral experiment assessing the effect of isochronous retiming of speech on speech perception in noise. Two types of anchor points were defined for retiming speech, namely syllable onsets and amplitude envelope peaks. For each anchor point type, retiming was implemented at two hierarchical levels, a slow time scale around 2.5 Hz and a fast time scale around 4 Hz. Results show that while any temporal distortion resulted in reduced speech intelligibility, isochronous speech anchored to P-centers (approximated by stressed syllable vowel onsets) was significantly more intelligible than a matched anisochronous retiming, suggesting a facilitative role of periodicity defined on linguistically motivated units in processing speech in noise.

## 1. Introduction

Human speech perception is remarkably robust to the auditory perturbations commonly encountered in everyday communicative situations, such as the babble noise in the background of a busy café. Indeed, under such conditions, recognition rates that can be achieved outstrip those attained by the best currently available automatic methods. While much is known about the role of spectral cues in recognizing speech in noise, and about how speech intelligibility is affected by energetic masking, much less is known about the role that temporal factors play.

Temporal factors are important for the comprehension process because perception of temporal properties underlies the human ability to focus attention on the target source and to segregate it from competing sources. Recently, interest in temporal mechanisms in speech recognition has been bolstered by proposals concerning the role of brain oscillations in sensory perception in general (Buzsaki and Draguhn, 2004) and speech processing in particular (Giraud and Poeppel, 2012b). Specifically, it has been proposed that cortical oscillations underpin mechanisms of sensory selection (Schroeder and Lakatos, 2009) and the parsing of important elements of the speech signal for further decoding (Giraud and Poeppel, 2012a).

Brain oscillations are fluctuations of local field potentials that can be measured using electrophysiological techniques and reflect the excitability of population of neurons. Increased excitability translates into increased dynamic range for coding information by those neurons ensembles. Although cortical oscillations arise due to basic physiological mechanisms (endogenous rhythms), it has been shown that these oscillations are not fixed (even single neurons can oscillate at different frequencies Hutcheon and Yarom, 2000); moreover, cortical oscillations entrain to the fluctuations of external stimuli (Ahissar et al., 2001; Lakatos et al., 2005, 2008; Aiken and Picton, 2008). It is this latter property that has motivated proposals that phases in the processing efficiency are interactively aligned to important phases in the unfolding of spoken information (Large and Jones, 1999; Schroeder and Lakatos, 2009; Besle et al., 2011; Peelle and Davis, 2012). Such a coupling would result in a net increase in processing efficiency for those parts of speech that are crucial to decode. This idea has received experimental support from findings that theta phase patterns track spoken sentences, with the degree of tracking correlated with speech intelligibility (Ahissar et al., 2001; Luo and Poeppel, 2007; Nourski et al., 2009; Peelle et al., 2013).

In addition to a general alignment of cortical and speech oscillations, there is an enticing link between the timing of cortical oscillatory activity and the duration of specific spoken linguistic units. For example, the time scale of delta-theta band activity (2–8 Hz) corresponds to the average frequency of the syllabic rate, i.e., in English stressed syllables occur at a rate below 4 Hz (Greenberg et al., 2003) and across languages the syllable rate is between 5 and 8 Hz (Pellegrino et al., 2011). Gamma band activity (25–70 Hz) has an approximate correspondence with the duration of subphonemic elements. Further, frequency nesting, or the dependence of higher frequency power on the phase of a lower frequency band is a pervasive phenomenon in brain oscillations (Canolty and Knight, 2010) and has consistently been observed between the delta and gamma band during speech processing (Schroeder and Lakatos, 2009; Ding and Simon, 2014). This hierarchical relationship bears a striking similarity to the nesting of linguistic units in speech, i.e., syllables are composed of phonemes, and words are composed of syllables (although strict inclusion of a smaller unit into a larger unit is not always the case, as seen, for example, in French syllabification). Taken together, these results have led researchers to propose that brain oscillations act as an active parser of speech, packaging the continuous stream of information into units of different granularity for further processing (Giraud and Poeppel, 2012a).

Although the linking of cortical oscillations and speech processing provides a rich framework for understanding the efficiency of speech perception, a number of important details remain unclear. For example, it is not clear which speech units and properties provide the basis for cortical tracking. The syllable has been proposed as a central unit with the critical-band amplitude envelopes playing a vital role (Greenberg et al., 2003; Luo and Poeppel, 2007; Ghitza, 2013; Ghitza et al., 2013). However, it should be noted that the syllable rate is not robustly encoded in the fluctuations of the amplitude envelope (Cummins, 2012). That is, although in most languages, and for carefully articulated forms of speech, a syllable consists of a vocalic nucleus in which the amplitude peak stands out in relation to surrounding consonants, this characterization does not always hold in the more commonly encountered conversational speech forms. This is due to reduction phenomena where vocalic nuclei can be deleted (Meunier and Espesser, 2011), and voiced consonants can be louder than the vocalic nucleus. Therefore, in continuous speech, the mapping of amplitude peaks to a syllable or stressed syllable is not as straightforward. Perhaps a more useful conceptualization of the speech cues that may be involved in driving cortical entrainment (and parsing) is that of P-centers, or perceptually defined moments of occurrence of word onsets (Morton et al., 1976). While P-centers bear a close connection with the syllabic description of an utterance, they may constitute an appropriate level of description for determining cortical entrainment to speech, in that they provide a perceptually grounded parsing of beats of an utterance.

Another uncertainty in connecting cortical oscillations and speech processing concerns whether the entrainment of cortical oscillations is driven primarily by the bottom-up physical characteristics of the stimulus or whether top-down control is important. On the one hand, oscillatory activity could be the result of a direct bottom-up response to the physical patterning of the stimulus, since the speech amplitude envelope has been found to correlate with the cortical response (Ahissar et al., 2001). On the other hand, the speech envelope is often obscured by noise (Houtgast and Steeneken, 1985), which would require an active phase resetting mechanism to realign cortical tracking (perhaps with salient acoustic events playing a role, Doelling et al., 2014). Moreover, it has been shown that entrainment still occurs even in the absence of amplitude fluctuations (Zoefel and VanRullen, 2015a) or marked auditory events signaling onsets of complex auditory rhythmic patterns (Chapin et al., 2010; Barczak et al., 2015) and that phase locking is affected by intelligibility even though stimuli have the same amplitude envelope (Peelle et al., 2013). These demonstrations have led some researchers to propose that top-down factors must be taken into account when considering the cortical tracking of speech (Obleser et al., 2012; Peelle et al., 2013).

In summary, the oscillation perspective is an important one but a number of prominent issues need to be clarified. The main issue we address in the current study concerns the basis for tracking the speech signal and can be summed up by this question: Is tracking based purely on a low-level physical property, such as amplitude envelope, or do top-down linguistic factors have a role to play? To test this we determined how timing modifications that imposed periodicity on naturally produced speech (i.e., making speech isochronous) affect intelligibility. Here, we manipulate the basis of timing modification and take intelligibility as an index of speech processing efficiency. Given the proposed link between intelligibility and cortical entrainment, we interpret the results in the context of the neurocognitive framework of brain oscillations.

In setting out to test the influence of imposed periodicity on speech perception we chose to examine speech perception in noise. One reason to test in noise was so that correct word identification performance would be well away from ceiling levels; so providing a chance to readily observe the effects of any manipulation. More importantly, testing such a manipulation in a noise environment is well motivated by theory. That is, although cortical tracking can be maintained in noise (Ding and Simon, 2013; Ding et al., 2014) it is likely that with an increasing noise level, more frequent phase resetting would be required due to the increased sparsity of available speech information and the concomitant increase in uncertainty about the speech sequence being processed. If there is some cost associated with this online adjustment, then a stimulus with isochronous periodic characteristics should attract less cost since its phase would be easier to predict. Here, the effect of isochrony on intelligibility would be greatest when the anchor point used for the temporal modification coincides with what is important for cortical tracking. Another possibility is that in the absence of predictable cues for phase resetting, the oscillatory system engages into a default set of “idle” frequencies at values typically observed during speech perception, which may provide a processing benefit when the high excitability phases align with important speech information.

In the current study, we transformed naturally timed speech to an isochronous form, and compared it with the baseline of unmodified speech and a matched transformed condition of anisochronous speech (see Section 2.2.1). To test a bottom-up account of cortical tracking we used peaks in the amplitude-envelope as anchor-points to render speech isochronous. That is, we hypothesized that if cortical entrainment is mainly driven by salient acoustic cues then making those cues regular should lead to intelligibility benefits in noise. To test whether cortical tracking used higher-level acoustic cues defined in linguistic terms, we used syllable onsets as anchor points for the isochronous modification.

We also assessed the effect of the isochronous modification at two levels of timing corresponding to two distinct frequencies. Entrainment has been observed in the delta-theta range spanning 2–8 Hz, which encompasses the average frequency of two hierarchical metrical levels in speech: that of the stressed syllable and that of the syllable. These two timing levels were chosen for the syllable-based transformation, and were matched in frequency for the amplitude envelope-based isochronous transformation.

Selecting the stressed syllable onset as an anchor point provides a way of operationalizing the concept of perceptual beat, or P-center, since the latter tend to be located near the onset of vowels in stressed syllables (Allen, 1972a,b; Morton et al., 1976). Note that in employing syllable and stressed syllable onsets as anchor points we are not necessarily proposing that oscillatory mechanisms need knowledge of syllable and stress boundaries but simply that these are sensitive to the perceptual beat or rhythm of an utterance.

In Section 2, we present the speech material used and detail the isochronous transformation, introducing a temporal distortion metric that defines the anisochronous transformation. Listeners results are presented in Section 3, and discussed in Section 4.

## 2. Materials and methods

### 2.1. Stimuli

One hundred and ninety sentences from the Harvard set (Rothauser et al., 1969) were spoken by a female native Australian English talker in her mid-twenties. The sentences had at least five keywords and were mildly predictable, such as in *Large size in stockings is hard to sell*. Sentences were individually segmented and automatically forced-aligned into words and phonemes. Phoneme boundaries were manually checked and corrected. Syllables were individually coded as stressed or unstressed based on a dictionary lookup^1^ of lexical stress and manually adjusted for sentence level stress patterns and particular production of the talker.

The amplitude envelope of speech was computed by taking the root mean square of the waveform amplitude values for adjacent 16 ms frames and the final value was taken as the running average over 7 frames. Peak values were selected iteratively by selecting the maximum of the envelope, marking the surrounding 80 ms to prevent from subsequent selection, and repeating the process until no maximum value with a surrounding region could be determined.

### 2.2. Experimental design

#### 2.2.1. Isochronous transformation

**Figure 1:**
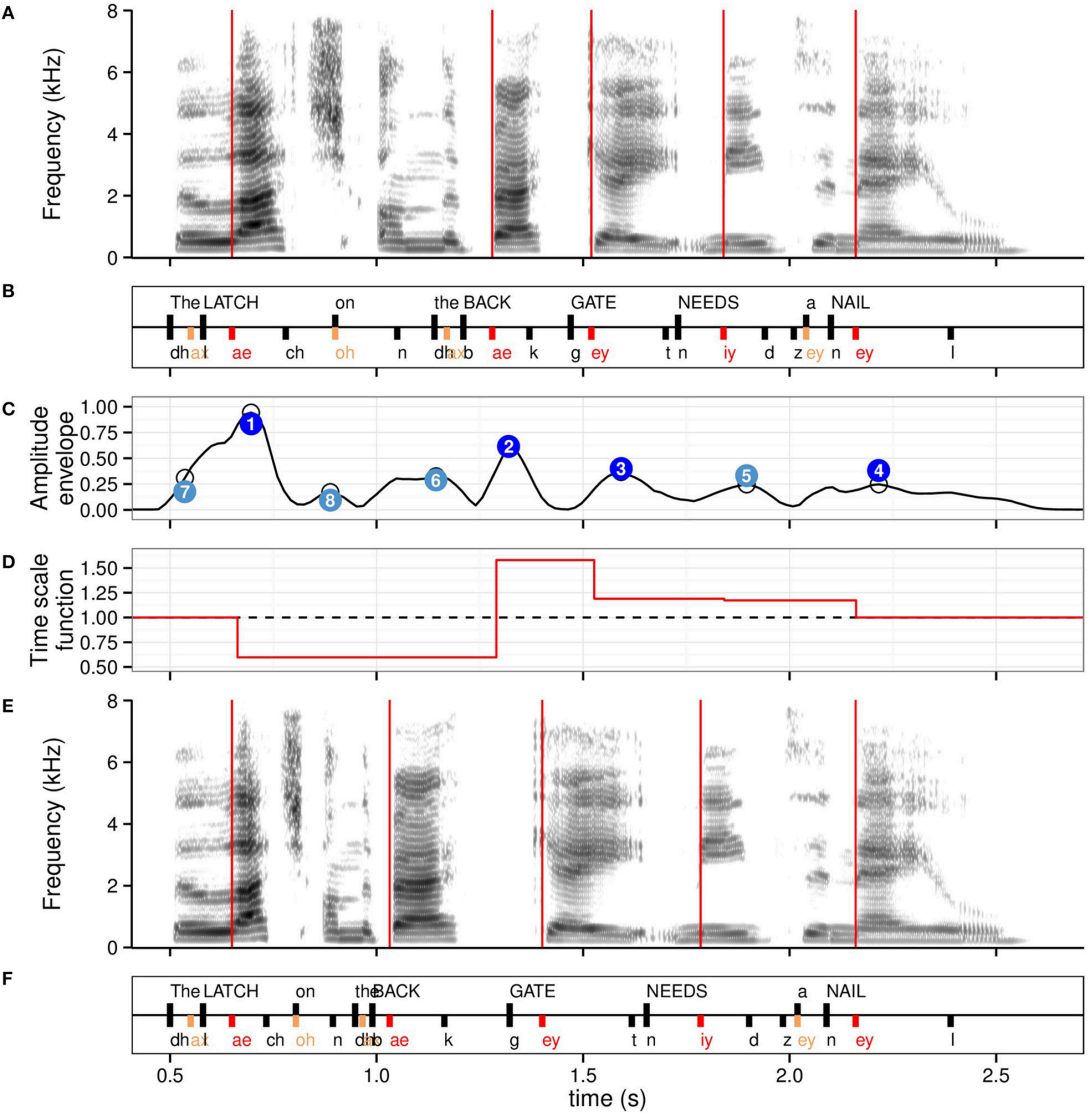
**Anchor points used for the isochronous modification, with the associated time scale function for the stressed syllable level for the example sentence ***The latch on the back gate needs a nail***. (A)** Spectrogram of the naturally timed sentence, with time instants of stressed syllables onsets overlaid in red. **(B)** Annotation in words (scored keywords in capital letters) and phonemes. Stressed syllables are in red, unstressed syllables in orange, remaining phonemes, and word onsets in black. **(C)** Amplitude envelope in normalized units with original peaks identified with empty circles. Peaks adjusted for sentence-level decay are shown in numbered circles with numbers indicating decreasing value order. Dark blue: four highest peaks; light blue: remaining peaks up to height. Note the relative agreement in timing between stressed syllables and low number of amplitude envelope peaks anchor points. **(D)** Time scale function for the stressed syllables anchor points. Values < 1 indicate compression, values >1 elongation. **(E)** Spectrogram of resulting isochronous sentence at the stressed syllable level, with time instants of isochronous stressed syllables onsets overlaid in red. **(F)** Resulting isochronous annotation.

The isochronous transformation (hereafter: *iso*) operates on each sentence by locally compressing or expanding contiguous speech regions so that these regions have an identical duration. For the *N* anchor points *a*_1_…*a*_*N*_ identifying the boundaries of speech regions in a sentence, the time scale function τ is defined as a step function that associates a time scale factor to each sample *n*:

(1) $$\begin{array}{l} \tau (n)=\{\begin{array}{ll} 1 & \text{if }n<a_{1}\\ \frac{a_{i+1}-a_{i}}{d} & \text{if }a_{i}<n\leq a_{i+1}\text{ with }1\leq i<N\;\\ 1 & \text{if }n\geq a_{N} \end{array} \end{array}$$

where $d=\frac{a_{N}-a_{1}}{N}$ is the mean duration of the sequence of speech regions to transform. With this definition, the timing of speech portions preceding the first anchor point and following the last anchor point remains unchanged, and so does the total duration of the speech regions. An example of the time scale function is seen in Figure 1D. The time scale factors are applied to the speech signal using WSOLA (Demol et al., 2005), a non-uniform time scaling algorithm that achieves high naturalness by adjusting local time scale factors according to sound class while preserving accurate timing.

We then define the temporal distortion metric δ which quantifies the amount of elongation and compression applied to a sentence, as the root mean square of the log-transformed time scale factors:

(2) $$\begin{array}{l} \delta =\sqrt{\frac{1}{N}\overset{N}{\underset{n=1}{\sum }}log{\left(\tau \left(n\right)\right)}^{2}}. \end{array}$$

This metric enables us to further define an anisochronous transformation (*aniso*) as a counterpart of the isochronous one, which applies identical amount of temporal distortion as the latter but which does not result in equal duration of speech regions. This condition is important in our design as it will help to disentangle the effects of periodicity and temporal distortion in performance. Here, the anisochronous transformation was operationally defined by applying the time scale factors obtained for the isochronous transformation in a reverse order, i.e., τ_*aniso*_[1, 2, …, *N*] = τ_*iso*_[*N, N* − 1, …, 1]. This way, anisochronously retimed anchor points will have locations which are perceptually unpredictable from neither the original nor the isochronously retimed anchor point locations.

#### 2.2.2. Anchor points

Two types of anchor points were defined to assess the effect of isochronous retiming: the *syllable* and the *amplitude envelope* anchor points. For each of the anchor point type, retiming was implemented at a slow and a fast time scale (syllable: *stressed* and *all syllables*; amplitude envelope: *low* and *high number of peaks*, respectively).

The stressed syllable anchor points (*str*) were taken as the onset of the nuclear vowel of stressed syllables in the sentence. This time point is considered to carry the perceptual beat of a syllable (Allen, 1975; Port, 2003) and is a good approximation of the P-center (Morton et al., 1976; Scott, 1993) which exact location varies as a function of the length of the consonant cluster of the syllable onset (Patel et al., 1999; Cummins, 2015). Using stressed syllables as anchor points for the isochronous transformation results in a form of speech that is similar to that obtained in a speech cycling task where talkers are asked to repeat a sentence in the presence of a regular timing beat (Cummins and Port, 1998). The all syllable anchor points (*syl*) were obtained by selecting vowel onsets of all syllables in the sentence.

These two hierarchical levels were matched for the amplitude envelope anchor points. For the current material of sentences with a mean duration of 2.10 s (*SD* = 0.25), low number of peaks (*loN*) anchor points were empirically selected as the first 4–5 peaks of the amplitude envelope. The exact number of peaks was chosen as the number of peaks that led to the least temporal distortion (see Equation 2). Similarly, high number of peaks (*hiN*) anchor points were selected as the first 7–8 highest peaks, whichever led to the least temporal distortion. Prior to peak selection, the amplitude decay that occurs naturally in production between the beginning and ending of a sentence was compensated for by applying a correction factor to the values of the envelope peaks. The decay was estimated by the slope *a* of the regression line modeling the amplitude values of the eight highest envelope peaks, and the value of the peaks were adjusted according to:

(3) $$\begin{array}{l} y_{i_{adj}}=y_{i}-at_{i}+a\frac{t_{N}-t_{1}}{2} \end{array}$$

where *y*_*i*_*adj*__ is the adjusted amplitude value of peak *i* (*i* = 1, …, *N*; *N* = 8), *y*_*i*_ is the amplitude value of peak *i* and *t*_*i*_ the time instant of peak *i*. The resulting regression line modeling the adjusted peak values has a null slope, and the resulting ordering of the adjusted peak values is normalized with reference to amplitude decay. Original and adjusted peak values are represented in Figure 1C.

Figure 1 shows the two types of anchor points at two hierarchical levels for an example sentence, along with the resulting isochronous retiming using the stressed syllable anchor points (condition *iso.str*).

**Figure 2:**
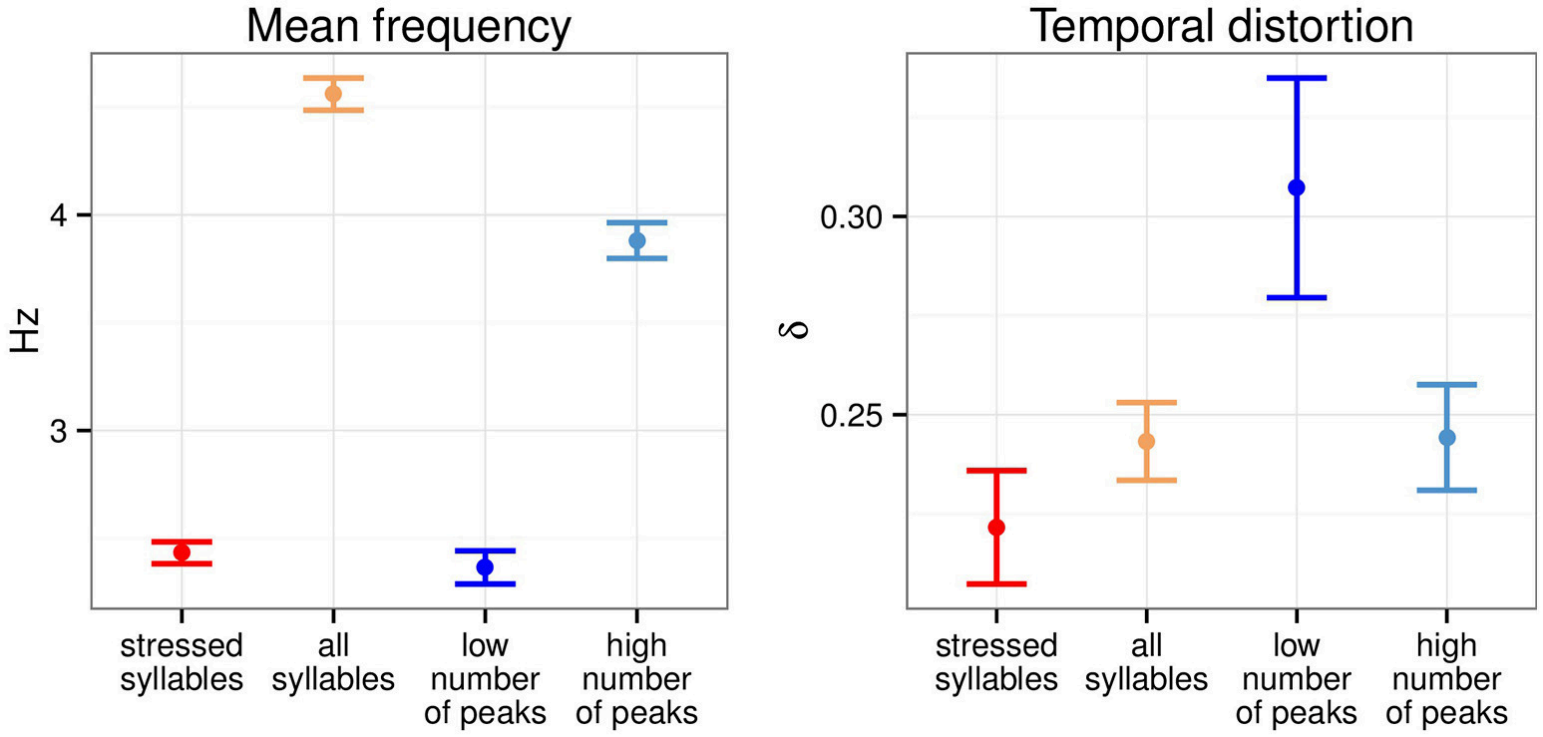
**Mean inter-anchor point frequency and temporal distortion across four temporal modification conditions**. Errobars, here and elsewhere, show 95% confidence intervals (*N* = 190).

Figure 2 summarizes the average inter-anchor point frequency and the temporal distortion across the four transformation conditions over the 190 sentences of the corpus. Low number of peaks anchor points had an average frequency of 2.37 Hz, not significantly different from stressed syllables anchor points (2.43 Hz). High number of peaks anchor points were paced at a lower frequency than all syllables anchor points (3.33 and 4.56 Hz respectively). Low number of peaks anchor points selection led to the greatest temporal distortion while stressed syllables anchor points were the one leading to the least temporal distortion. Both high number of peaks and all syllables anchor points led to similar temporal distortion.

**Figure 3:**
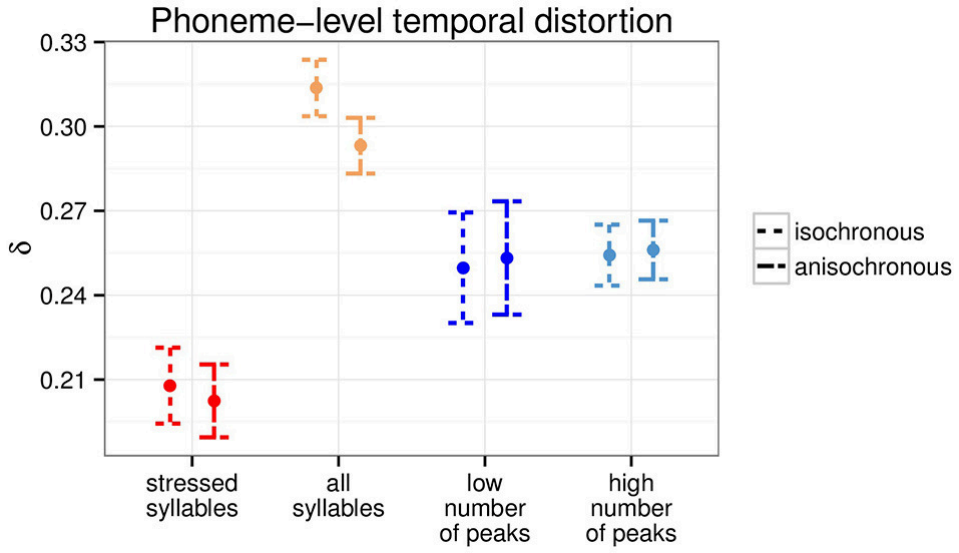
**Phoneme-level temporal distortion for the two anchor point types at the two time scales (***N*** = 190)**.

The two types of anchor points being different in nature, with one being linguistically grounded and the other signal based, there is the possibility that they affect the timing of phonetic segments in different ways. We examined the phoneme-level temporal distortion by computing the temporal distortion (Equation 2) over successive phoneme units instead of every sample. Figure 3 shows the phoneme-level temporal distortion for the two types of anchor points. Both isochronous and anisochronous modifications led to comparable phoneme-level temporal distortion [all *p* > 0.5, for individual Welch two sample *t*-tests per anchor point type], to the exception of the all syllable level, where the isochronous modification resulted in significant greater phoneme-level temporal distortion than the anisochronous one [*t*_(377.93)_ = 2.85, *p* < 0.01].

The effect of the two types of anchor points were tested in two separate experiments. Experiment I tested the effect of the syllable based anchor points and Experiment II that of the amplitude envelope based anchor points. Both had five conditions: an unmodified naturally timed speech condition (*unmod*) and four other conditions obtained by crossing the transformation polarity with the two metrical levels of the anchor points (Experiment I: *unmod, iso.str, aniso.str, iso.syl, aniso.syl*; Experiment II: *unmod, iso.loN, aniso.loN, iso.hiN, aniso.hiN*). Example stimuli can be found in Supplementary Materials.

### 2.3. Participants and procedure

Participants were recruited from the undergraduate population of Western Sydney University and through personal acquaintances. University students received course credit for participation while other participants did not receive any remuneration. All participants provided informed consent and reported normal hearing. All research procedures were approved by the Human Research Ethics Committee of Western Sydney University under the reference H9495. Thirty participants took part in Experiment I. Four participants were discarded following performance-based exclusion criteria detailed in Section 2.3.1, leaving twenty-one females and five males with mean age of 20.9 (*SD* = 6.3). A different cohort of thirty participants were recruited for Experiment II and one participant was excluded following the same exclusion criteria as in Experiment I, leaving twenty-four females and five males with mean age of 20.9 (*SD* = 5.9) for analysis.

Participants were tested individually and sat in a sound attenuated booth in front of a computer screen, where there were presented with online instructions. Both experiments had an identical setup: sentences mixed with noise were presented in blocks and the participants had to type what they heard. The experiments were self-paced, and participants could take a break after the third block out of five. Stimuli were presented over BeyerDynamic DT 770 Pro 80 Ohm closed headphones at a fixed level. Sentences were mixed with speech-shaped noise (SSN) at a fixed signal-to-noise ratio of −3 dB SNR. SSN was constructed by filtering white noise with 200 LPC coefficients taken from the long-term average speech spectrum computed on a concatenation of all sentences of the corpus. RMS energy of sentence-plus-noise mixtures were individually adjusted to a fixed value of 0.04. Each experiment took 45 min to complete on average.

In both experiments, sentences were blocked in five sets of thirty-six sentences. Block order was determined by a latin square design. Sentences were randomly distributed across the five conditions for each participant so that each participant heard each sentence only once and each sentence could be heard in different condition across participants. Within each block, sentences were ordered from low to high anchor points frequency, in order to minimize perceived rhythmic change from trial to trial. The remaining ten sentences were presented as practice, two at the beginning of each block, and were not used for scoring.

#### 2.3.1. Scoring

In both experiments, typed sentences were scored by counting the correct keywords per sentence. Keywords were determined from the sentence orthographic form by excluding a list of function words, such as “a,” “the,” “for,” “in.” Original orthographic sentence and typed responses were parsed into a canonical form to account for homophones and spelling mistakes, and the proportion of matching words was computed for each typed sentence.

Data from participants who scored less than 20% in at least one condition or did not provide a response for at least 50% of the sentences across any condition were discarded from the dataset.

## 3. Results

### 3.1. Listeners' performance

**Figure 4:**
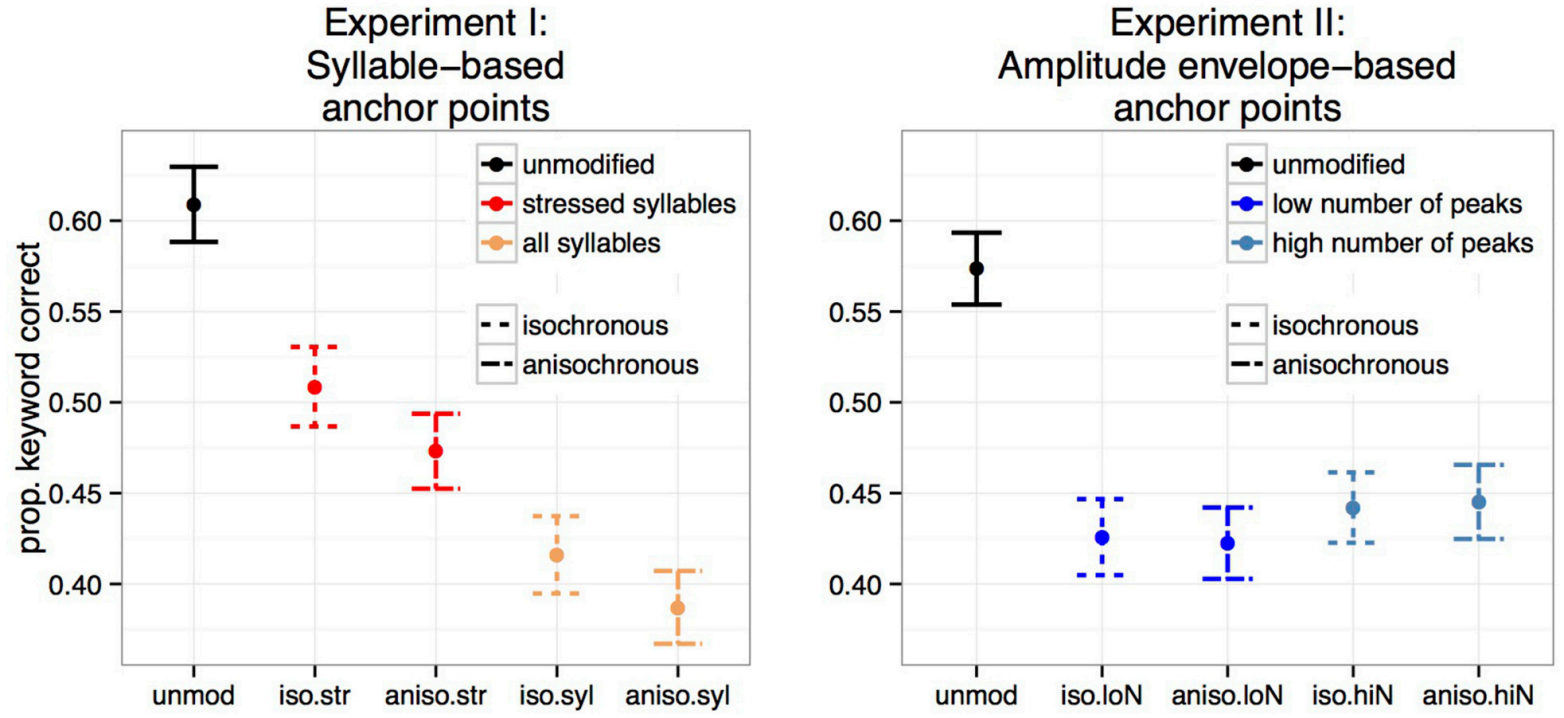
**Mean proportion of correctly identified keyword per sentence over participants for the five conditions**. Experiment I: syllable-based anchor points (*N* = 26). Experiment II: amplitude envelope-based anchor points (*N* = 29).

Figure 4 shows the proportion of keywords correctly identified for Experiments I and II. Temporally unmodified speech in noise was best recognized in both experiments. Stressed-syllable based retimed speech was better recognized than all-syllable based retimed speech (Experiment I) while both metrical levels had similar intelligibility reduction in Experiment II. Crucially, isochronous retiming led to better recognition for syllable based transformations (Experiment I) but not for amplitude based ones (Experiment II).

For each experiment, we evaluated the effect of the five conditions on intelligibility with a generalized linear mixed model applied to individual word counts. We used *condition* with five levels as the fixed effect, and intercept for subjects and sentence as random effects. *P*-values were obtained by conducting simultaneous tests for general linear hypotheses, specifying a matrix of contrast across the conditions (function glht() of the *lme4* R package, Bates et al., 2015). Random effects standard deviation of subject and sentence were 0.40 and 1.10 respectively for Experiment I and 0.36 and 1.10 respectively for Experiment II. Results of individual comparisons are given in Table 1 for both experiments.

**Table 1:** **Output of generalized linear mixed models fitted separately for each experiment**.

***n***	**Experiment I**					**Experiment II**				
	**Comparison**	**Estim**.	***z***	***p***	**Sig**.	**Comparison**	**Estim**.	***z***	***p***	**Sig**.
1	*iso.str, unmod*	−0.545	−12.16	< 0.001	^***^	*iso.loN, unmod*	−0.847	−19.87	< 0.001	^***^
2	*aniso.str, unmod*	−0.722	−16.06	< 0.001	^***^	*aniso.loN, unmod*	−0.821	−19.18	< 0.001	^***^
3	*iso.syl, unmod*	−1.017	−22.40	< 0.001	^***^	*iso.hiN, unmod*	−0.713	−16.78	< 0.001	^***^
4	*aniso.syl, unmod*	−1.127	−24.68	< 0.001	^***^	*aniso.hiN, unmod*	−0.666	−15.67	< 0.001	^***^
5	*iso.str, aniso.str*	0.177	4.00	< 0.001	^***^	*iso.loN, aniso.loN*	−0.027	−0.63	0.965	
6	*iso.syl, aniso.syl*	0.110	2.43	0.092	.	*iso.hiN, aniso.hiN*	−0.047	−1.12	0.771	
7	*iso, aniso*	0.287	4.54	< 0.001	^***^	*iso, aniso*	−0.074	−1.24	0.700	
8	*syl, str*	−0.878	−13.80	< 0.001	^***^	*hiN, loN*	0.290	4.82	< 0.001	^***^

*Each numbered line shows a comparison, its estimate, the z-value and associated p-value, and visual indication of significativity*.

As shown by comparisons 1–4 in Table 1, all transformed speech conditions resulted in significantly poorer intelligibility than unmodified speech, for both experiments. Next, testing the isochronous modification against the anisochronous modification separately for each anchor point type revealed that when stressed syllables are taken as anchor points, isochronous speech is more intelligible than anisochronous speech (Experiment I, comparision 5). A tendency for this effect is observed when all syllables are taken as anchor points (Experiment I, comparision 6). In contrast, when applied to anchor points defined on the amplitude envelope, the isochronous transformation did not result in intelligibility changes, for any of the low and high number of peaks (Experiment II, comparisions 5 and 6 respectively). This effect was also observed when collapsing identical anchor point types within each experiment: in Experiment I, isochronous speech was more intelligible than anisochronous speech regardless of the anchor point type (Experiment I, comparision 7) and in Experiment II, intelligibility of isochronous speech was not distinguishable from intelligibility of anisochronous speech when collapsing across anchor point types (Experiment II, comparision 7). Finally, in both experiments, the choice of anchor points had a clear net effect on intelligibility, with transformations anchored on stressed syllable being significantly more intelligible than that implemented on all syllables (Experiment I, comparision 8), and transformations anchored on high number of peaks being slightly but significantly more intelligible than transformations anchored on low number of amplitude envelope peaks (Experiment II, comparision 8).

### 3.2. Sentence intelligibility

As shown in Figure 2, the choice of anchor point type resulted in marked differences in inter-anchor points frequency and temporal distortion. We examined the relation of these metrics with transformed sentences intelligibility. Unmodified sentences were not analyzed as these metric do not apply to them. Table 2 shows the correlation between transformed sentences intelligibility and temporal distortion on one hand, and with mean frequency on the other hand.

**Table 2:** **Correlation between intelligibility scores of transformed sentences and ***a***. Temporal distortion; ***b***. Mean frequency for both Experiments across all subjects**.

**Experiment I**					**Experiment II**				
**Anchor point**	**Transf**.	***r***	***p***	**Sig**.	**Anchor point**	**Transf**.	***r***	***p***	**Sig**.
**a. Temporal distortion**									
Stressed syllables (*str*)	*iso*	−0.19	0.000	^***^	Low number (*loN*)	*iso*	−0.39	0.000	^***^
	*aniso*	−0.20	0.000	^***^		*aniso*	−0.31	0.000	^***^
All syllables (*syl*)	*iso*	−0.26	0.000	^***^	High number (*hiN*)	*iso*	−0.25	0.000	^***^
	*aniso*	−0.31	0.000	^***^		*aniso*	−0.22	0.000	^***^
**b. Mean frequency**									
Stressed syllables (*str*)	*iso*	−0.03	0.382		Low number (*loN*)	*iso*	0.20	0.000	^***^
	*aniso*	0.03	0.439			*aniso*	0.18	0.000	^***^
All syllables (*syl*)	*iso*	0.15	0.000	^***^	High number (*hiN*)	*iso*	0.23	0.000	^***^
	*aniso*	0.25	0.000	^***^		*aniso*	0.24	0.000	^***^

*Each cell displays the anchor point type, the polarity of the transformation, the Pearson's product moment correlation coefficient with its associated p-value, and a visual indication of significativity*.

Table 2a shows that transformed sentences intelligibility was negatively correlated with temporal distortion. This correlation applied across the board for all transformation conditions, with a highest value for low number of peaks isochronous, a condition that had highest temporal distortion, and that also lead to a low intelligibility score. Within an identical anchor point type condition, isochronous and anisochronous transformed sentences had similar correlation coefficients, probably owing to the fact that sentences did indeed have identical distortion factors.

Also displayed in Table 2b is the result that retimed sentences' mean frequency was positively correlated with sentence intelligibility, with a notable exception of sentences that were retimed according to stressed syllables, where mean retimed sentence frequency did not explain any intelligibility variation.

Further analysis was conducted to evaluate the correlation between temporal distortion and mean frequency of retimed sentences. Only isochronous sentences were analyzed as anisochronous sentences have identical temporal distortion and mean frequency to isochronous ones. A negative correlation for sentences retimed using all syllables as anchor points was found (*r* = −0.28, *p* < 0.001) and similarly with any of the amplitude envelope-based anchor points (low number of peaks: *r* = −0.33, *p* < 0.001; high number of peaks: *r* = −0.51, *p* < 0.001). However, no correlation was found for sentences retimed at the stressed syllable level (*p* = 0.25).

## 4. Discussion

In this study, we evaluated the effect on speech intelligibility of imposing isochronous timing on sentences presented in stationary noise. The idea that isochronous timed speech may be more intelligible in noise is based on the proposal that when speech information is degraded, an isochronous rhythm will make the tracking of important speech time instants, identified here as anchor points, more reliable.

Two approaches for implementing isochronous transformations were contrasted. The first used linguistically defined anchor points, namely, stressed syllables, and all syllables; the second used amplitude envelope peaks with the number of peaks matched to the frequency range of stressed syllables (low number of peaks: 4–5 peaks, approx. 2.5 Hz) and all syllables (high number of peaks: 7–8 peaks, approx. 4 Hz, see also Figure 2).

We found that sentences where the isochronous retiming used stressed syllables as anchor points were significantly more intelligible than those having a matched anisochronous transformation, and we also observed a tendency for a benefit from the use of all syllables anchor points. In contrast, isochronous transformations based on the peaks in the speech amplitude envelope had no effect on intelligibility. Before discussing these differential intelligibility benefits, we will first consider the finding that any departure from the natural speech timing resulted in a decrease of intelligibility.

### 4.1. Intelligibility decrease for retimed speech

The result that retimed speech was less intelligible than naturally timed speech in stationary noise accords with the results of a previous study of speech intelligibility using nonlinear retimed speech (Aubanel and Cooke, 2013). However, it does appear to be at odds with the idea that when the speech signal is degraded by noise, isochronous timing between the important speech regions should promote processing efficiency. That natural speech is best recognized can be explained when considering speech perception processes as broadly divided into two stages, as in the Tempo model proposed by Ghitza (2011). In this approach, the first stage involves the registration and perception of the speech signal, the second stage involves the use of that information to access stored representations. Under this scheme, we suggest that in noise, isochronous syllable-based timing assists in the first stage, but that any advantage gained over natural speech is more than offset by the advantage that natural timing of speech has in the second stage. In what follows, we consider processing at each stage in turn.

In regards to the first stage of processing, it was found that compared to an anisochronous control, an imposed isochrony boosted intelligibility only when the retiming anchor points occurred at the onset of the stressed syllable. Here we propose that while the oscillatory tracking system is highly flexible, its ability to phase reset to the salient properties of ongoing speech breaks down under energetic masking conditions. In the absence of continuity cues, one interpretation is that cortical oscillations revert to a default state with an “idle” fixed frequency in the theta range typical when listening to speech. When the phase of this oscillatory mechanism aligns with regularly occurring stressed syllable onsets, increased processing efficiency results in intelligibility increase.

The finding that stressed syllable onsets are an important temporal cue in English speech perception fits with theories of speech processing that highlight the importance of the syllabic level (Greenberg et al., 2003; Luo and Poeppel, 2007; Giraud and Poeppel, 2012a; Peelle and Davis, 2012; Ghitza, 2013). Indeed, converging results from different fields of research point to the importance of syllable onsets in speech perception, e.g., the onset timing of vowels is mostly preserved in spontaneous compared to laboratory speech (Greenberg, 1999), the perceptual beat associated with stressed syllable, or P-center, is located in the vicinity of the syllable onset and supports meter representation (Port, 2003). Given the results of the current study, one could hypothesize that cortical oscillations track P-centers, despite the fact that the latter do not striclty align with salient acoustic cues such as amplitude envelope peaks.

It is in regard to the second stage of processing for which we propose that the advantage for natural timed speech accrued. That is, naturally timed utterances have an advance at the recognition stage where spoken representations are accessed. This is because processing at this stage makes reference to the listener's knowledge about timing statistics learned through exposure to spoken language, and used in production. This knowledge of regular timing patterns enables listeners to make precise and minute predictions about the ongoing speech stream (Pickering and Garrod, 2013). However, in the case where speech has been retimed (isochronously or not) the signal will not match these predictions and recognition will suffer. This notion that a mismatch between the input and an expected timing profile impairs recognition is supported by the current analysis showing that intelligibility correlates negatively with the amount of temporal distortion applied to the sentences.

It is the nature of the temporal distortion implemented here, with applies an alternation of compression and expansion within the same sentence, that may be most harmful for breaking timing expectations. Uniform transformations to speech timing are less detrimental to recognition as the perceptual system is thought to adjust future predictions based on the ongoing speaking rate. As Dilley and Pitt (2010) showed, the identification of a speech target depends on the speaking rate of the preceding context, and a mismatch between the speaking rate of the target and preceding context leads to the reinterpretation of the target to match the preceding context.

It should be noted here that the timing statistics which together make up the speech rhythm of a particular language do not usually result in periodicity. Similarly, the literature does not posit a fixed frequency for oscillation but a frequency range. If periodicity were a necessary dimension of successful speech communication then talkers would use this form of speech. On an informational theoretic angle, microvariation in timing also allow the encoding of information—a perfectly regular “carrier”-like speech rhythm would be impoverished in information, and would limit suprasegmental encoding. We instead propose that periodicity could have a facilitatory effect that would be exploited in situation where the input is corrupted by noise, and the minute temporal adjustements to track the ongoing speech stream is impaired.

The idea that in noise, cortical sampling falls back to a default sampling period has interesting implications for the design of a follow-up study. In the current one, the period of isochrony was derived from the average occurrence of anchor points, which, for stressed syllables, ranged from 2.38 to 2.48 Hz (see Figure 2). Sentences were presented in increasing order of inter-anchor point frequency but no explicit indication of this frequency was given, nor was any explicit indication of the beginning of the first period provided. That is, no attempt was made to manipulate initial phase resetting or subsequent phase tracking and indeed, none of the participants reported periodicity in the stimuli. Given this, one could hypothesize that providing explicit cues for onset and periodicity may result in a greater intelligibility increase for isochronous timing, potentially due to increased alignment between brain oscillations and stimulus characteristics.

### 4.2. Relative contribution of syllabic information vs. amplitude envelope to cortical tracking

In addition to finding support for isochrony anchored to the stressed syllable (Experiment I), an important finding was that the isochronous modification anchored to the peaks in the amplitude envelope did not improve intelligibility compared to anisochronous retiming (Experiment II). At a methodological level, this null result confirms the validity of the anisochronous modification (i.e., reversing the polarity of any retiming does not in itself degrade intelligibility) and provides additional support for the positive result obtained for the linguistically based modifications. Note that we do not directly compare the listeners' performance across Experiment I and II as they used different cohorts of participants and had different intelligibility baselines for unmodified speech.

The null result in Experiment II is interesting in the light of the many studies that have proposed that the fluctuations of the amplitude envelope of speech play a crucial role in driving cortical entrainment (Ahissar et al., 2001; Luo and Poeppel, 2007; Aiken and Picton, 2008; Giraud and Poeppel, 2012a). In this regard, we point out that our results may be specific to the timing of important information in speech recognition in noise and as such do not call into question the importance of the amplitude envelope for speech recognition in general.

Both syllable and amplitude envelope based transformations led to modifications to the timing of the amplitude envelope. But while the time instants of amplitude envelope peaks were derived from the controlled retiming of syllable onsets in the syllable based retiming conditions, they were directly manipulated in the amplitude envelope retiming conditions. Interestingly, this direct control of the timing of amplitude envelope peaks did not reveal an isochronous advantage, suggesting that if periodicity may facilitate processing through alleviating the need of phase resetting, then amplitude envelope peaks were not the appropriate cues for phase resetting. In fact, it would be more correct to conclude that regularly occurring amplitude peaks misinform upcoming predictions, at least as much as irregular ones.

More specifically, it seems more plausible that amplitude envelope peaks act as second-order temporal cues in signaling neighboring linguistically meaningful units, and that it is the latter that constitute the primary temporal cues for speech recognition, and the support for phase resetting. This result is all the more relevant considering that amplitude envelope peaks are more more audible than syllable onsets in the type of speech shape noise employed here, and therefore the periodicity should be more readily accessed by the oscillatory system if it would be purely acoustically driven. In all, the results of this study support an increasingly shared view that if oscillations track incoming speech, top-down linguistic cues may play a stronger role than bottom-up acoustic cues (Obleser et al., 2012; Gross et al., 2013; Zoefel and VanRullen, 2015b; Ding et al., 2016).

Amplitude envelope peaks are usually assumed to constitute the critical time instants for amplitude envelope tracking, for example, in Ghitza (2012), the location of audible pulses indicating rhythm are placed at amplitude peaks. Our results nuance that account, indicating that P-centers, which are perceptually defined and linguistically informed temporal cues, could constitute a more appropriate level of description of the cues that drive cortical entrainment, at least when speech is presented in noise. While we contrasted two types of cues in the current study, other cues such as consonantal onsets or amplitude acceleration could provide further insight in the nature of the cues that support tracking.

English (including the Australian variety studied here) is traditionally considered as a stress-timed language as opposed to syllable- or mora-timed languages, although this distinction, which classically makes a hypothesis of constant duration of the corresponding units, has not been robustly verified empirically (Lehiste, 1977; Dauer, 1983; White and Mattys, 2007; Nolan and Jeon, 2014; Cummins, 2015). Nevertheless, in our study, isochronous retiming based on stressed syllable was the transformation resulting in the minimum temporal distortion, and also the condition that led to greater intelligibility compared to an all-syllable isochronous retiming. Apart from providing a weak support for stressed-syllable based isochrony in English, this result promotes the hypothesis that for syllable-timed languages, an opposite pattern of isochronous retiming benefit may be observed, with a greater benefit for isochronous retiming at the all-syllable level as opposed to higher metrical units.

A final point is that the current study employed behavioral measures of intelligibility to assess the effect of imposed periodicity in noise. A future line of research will be concerned with the evaluation of electrophysiological measures associated with this type of isochronous stimuli.

## Author contributions

VA, CD, and JK designed research; VA performed research; VA analyzed data; VA, CD, and JK wrote the paper.

### Conflict of interest statement

The authors declare that the research was conducted in the absence of any commercial or financial relationships that could be construed as a potential conflict of interest.

## Footnotes

^1^http://dictionary.cambridge.org/dictionary/british/
